# Underlying Chronic Lymphocytic Leukemia in a Case of Vivax Malaria With Thrombocytopenia: A Case Report

**DOI:** 10.7759/cureus.87363

**Published:** 2025-07-06

**Authors:** Amitabha Saha, Madhusha Mukhopadhyay, Arjun Talapatra, Tapas Bandhopadhay, Souryadeep Sardar

**Affiliations:** 1 Critical Care, Desun Hospital, Kolkata, IND; 2 Internal Medicine, Medicover Hospitals, Navi Mumbai, IND; 3 Critical Care Medicine, Manipal Hospitals, Kolkata, IND; 4 Internal Medicine, Manipal Hospitals, Kolkata, IND; 5 Medicine and Surgery, KPC Medical College and Hospital, Kolkata, IND

**Keywords:** leukemia, leukocytosis, malaria, pancytopenia, thrombocytopenia

## Abstract

Thrombocytopenia is a very commonly known complication of malaria. A 64-year-old male presented with high-grade fever, extensive petechial rashes, subconjunctival hemorrhage, and mucosal oral bleeds. Laboratory tests revealed severe thrombocytopenia (platelet count: 15,000 per cubic millimeter), leukocytosis (total leukocyte count (TLC): 29,100 per microliter), and coagulopathy (international normalized ratio: 4.2). Infection was ruled out by negative cultures. The smear was positive for *Plasmodium vivax*. Flow cytometry was done, and a diagnosis of chronic lymphocytic leukemia (CLL) was made. The association of leukocytosis with thrombocytopenia in the case of malaria, in the absence of sepsis - as in our patient - should raise a suspicion of underlying CLL.

## Introduction

Malaria is a common tropical disease, especially in Southeast Asian countries. Thrombocytopenia is a known manifestation of malaria, but in the absence of significant bleeding, it is not regarded as a defining clinical manifestation of severe malaria. In most cases, thrombocytopenia in malaria is not associated with bleeding and requires no treatment, with the platelet count rapidly returning to normal after successful treatment of the malarial episode [1]. Very severe thrombocytopenia, defined as fewer than 20,000 platelets per microliter, is an even rarer occurrence that has only been reported in a handful of cases [2]. Malaria is very commonly associated with chills, encephalopathy, and abdominal discomfort.

It is important to keep in consideration the various possible manifestations of *Plasmodium vivax*, as doing so may lead to the initiation of early, potentially life-saving therapy. Many studies have suggested that the paradigm of severe malaria is shifting, with one reporting that 32.7% of cases in the sample population exhibited severe characteristics when infected with *P. vivax* [3]. This highlights the importance of further investigating the complications associated with *Plasmodium malariae*.

More recently, low platelet counts have been associated with mortality in patients with *Plasmodium falciparum* and *P. vivax* infections [4]. In a study conducted by Das et al., it was found that anti-malarial drugs form reactive oxygen species (ROS) and cause lipid peroxidation, leading to cell death and lysosomal disruption, which can facilitate the killing of underlying chronic lymphocytic leukemia (CLL) cells [5]. Only a few anecdotal reports have been published regarding the incidental diagnosis of underlying CLL in malaria patients presenting with low platelets, which makes our study even more pertinent and relevant at present.

## Case presentation

A 64-year-old, non-comorbid male patient presented to our hospital with complaints of high-grade fever and lethargy for the past three days. Physical examination showed the presence of extensive petechial rashes, subconjunctival hemorrhage, and mucosal oral bleeds. The smear was positive for *P. vivax*. Laboratory tests revealed severe thrombocytopenia (platelet count: 15,000 per cubic millimeter), leukocytosis (total leukocyte count (TLC): 29,100 per microliter), and coagulopathy (international normalized ratio: 4.2). Bacterial infection was ruled out by a thorough clinical examination, negative blood cultures, and other negative infective markers like C-reactive protein (CRP) and procalcitonin. The patient was started on artesunate combination therapy at 2.4 milligrams per kilogram of body weight, as per protocol. His platelet count continued to worsen (5,000 per cubic millimeter) over the next three days. He was transfused with packed red blood cells (PRBCs), fresh frozen plasma (FFP), and platelets (random donor platelets (RDPs) and single donor platelets (SDPs)) as needed during this period. On the third day, we started a course of IV immunoglobulin, to which he responded favorably. His platelet count improved (100,000 per microliter), along with a sharp, significant increase in white blood cell (WBC) count (72,000 per microliter) over the following five days. Peripheral smear revealed smudge cells. This raised suspicion, and a hematology opinion was sought, and flow cytometry was performed. The laboratory investigations are presented in Table 1.

**Table 1:** Laboratory Investigations

Date	Hemoglobin (g/dL)	Packed Cell Volume (%)	Total Leukocyte Count (WBC/mcL)	Platelet (p/mcL)	Neutrophil (%)	Lymphocyte (%)	Sodium (mEq/L)	Potassium (mEq/L)
Reference Values	14-17	40%-54%	4,000-11,000	150,000-450,000	40%-60%	20%-40%	135-145	3.5-5.2
October 11, 2022	10.3	32.1%	31,160	7,000	46%	51%	139	4.1
October 12, 2022	8.8	26.9%	18,040	5,000	25%	74%	147	4
October 13, 2022	7.4	22.9%	25,400	10,000	27%	77%	153	4.1
October 14, 2022	7.6	23.5%	31,610	13,000	18%	81%	159	4.2
October 15, 2022	7.7	24%	50,400	25,000	20%	75%	161	4.7
October 16, 2022	7.2	23.4%	59,020	50,000	22%	70%	161	4.5
October 17, 2022	7.2	22.6%	61,830	70,000	15%	80%	161	4.2
October 18, 2022	8.3	26.8%	71,910	100,000	19%	80%	158	3.9
October 19, 2022	8.6	28%	99,710	136,000	20%	78%	156	3.4

**Figure 1:**
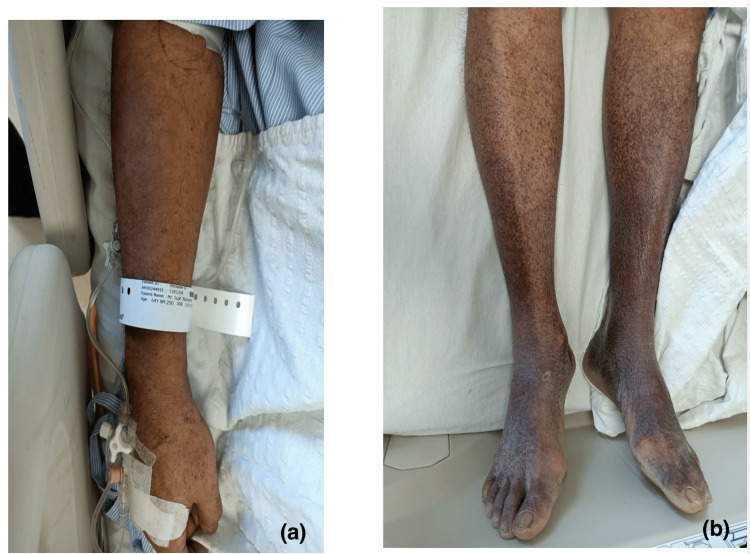
Patient Images (a) Small bleeding points seen over the upper limb, and (b) purpuric changes observed over the lower limbs.

Flow cytometry showed positive CD5, CD19, and CD23, suggesting the presence of mature B-cell CLL. The patient was started on oral prednisolone. Due to the severity of malaria, the artesunate dose was appropriately increased, which was subsequently changed to an artemether-lumefantrine combination once the patient stabilized. Primaquine was added at discharge at a calculated dose of 3.5 milligrams per kilogram of body weight, as per protocol, along with a tapering dose of prednisolone, with a plan for follow-up with the hemato-oncology team for further management (Figure 1).

## Discussion

*P. vivax*, the causative agent of vivax malaria, is the second most common species of malaria, with a yearly estimate of 35 million cases worldwide [6]. Thrombocytopenia (low platelet count) and leukocytosis (high WBC count) can be indicators of sepsis, a life-threatening condition caused by a dysregulated host response to infection. While leukocytosis is often associated with sepsis, thrombocytopenia can be a sign of sepsis or a complication that worsens the severity of the illness.

Pancytopenia in malaria patients leading to a diagnosis of CLL, acute lymphoblastic leukemia (ALL), or hemophagocytic lymphohistiocytosis (HLH) is documented in the literature [7,8]. In a study carried out at ESIC Model Hospital, Indore, Madhya Pradesh, out of 130 cases detected with vivax malaria, 100 cases had thrombocytopenia, while only 30 (13.04%) cases had a normal platelet count [9]. However, thrombocytopenia with leukocytosis in a malaria patient without sepsis can be a rare diagnostic challenge. The diagnosis can be confirmed by peripheral smear, flow cytometry, or bone marrow examination. In an endemic country like India, it is important to keep this diagnosis in mind while treating elderly patients with malaria. Further case reports and observational studies are required to validate the link between malaria and CLL.

There is a significant positive association between leukopenia and *P. falciparum* malaria. Leukopenia and thrombocytopenia are seen more commonly with *P. falciparum* malaria. Leukopenia occurs due to bone marrow suppression, resulting from cytokine production and/or folate deficiency when associated with pancytopenia. However, leukocytosis and thrombocytopenia are strong indicators of secondary bacterial infections in *P. falciparum* malaria [10]. Though leukopenia is commonly found in malaria, the presence of leukocytosis is unusual, especially in vivax malaria. This should raise a strong suspicion for an alternative diagnosis. Thus, *P. vivax* infection with leukocytosis raises various possibilities, and it becomes important to rule out underlying malignancies like CLL.

## Conclusions

Thrombocytopenia is a well-known effect of malaria, but is usually associated with leukopenia. The association of leukocytosis in the case of malaria, in the absence of sepsis - as in our patient - should raise a suspicion of underlying CLL. This aspect could be researched and studied more thoroughly in the future and could improve prognosis, leading to more prompt and successful treatment of CLL.
